# Supplementary material for: Are carbon emissions trading and green financial instruments synergistic? -Comprehensive quantitative research based on content analysis
Source: PLoS One. 2024 Mar 7;19(3):e0298601. doi: 10.1371/journal.pone.0298601 (PMC10919601; doi:10.1371/journal.pone.0298601)
Supplement: S1 Data — (ZIP) [file pone.0298601.s001.zip › Data/地级市Z_F工作报告.zip!/░▓╗╒/┬φ░░╔╜╩╨/└┤╫╘╣1⁄2╓┌║┼í╛┬φ┐╦╩2╛▌═°í┐_2008.docx]

# **政府工作报告（2008）**

【字体大小：[大](http://zwgk.mas.gov.cn/openness/detail/content/javascript:doZoom(18)) [中](http://zwgk.mas.gov.cn/openness/detail/content/javascript:doZoom(15)) [小](http://zwgk.mas.gov.cn/openness/detail/content/javascript:doZoom(14))】

——2008年1月5日在马鞍山市第十四届人民代表大会第一次会议上

市 长   姚玉舟

各位代表：
     现在，我代表市人民政府向大会作工作报告，请予审议，并请市政协委员和其他列席人员提出意见。
    一、过去五年工作回顾
     2003年市十三届人大一次会议以来的五年，是马鞍山发展史上很不平凡、具有特殊意义、取得巨大成就的五年。五年来，在省委、省政府和市委的坚强领导下，在市人大、市政协的监督支持下，我们坚持以邓小平理论和“三个代表”重要思想为指导，全面落实科学发展观，团结和带领全市人民，积极贯彻中央宏观调控政策，克服要素资源约束带来的压力，战胜多种突发公共事件造成的困难，全力推进经济、政治、文化和社会建设，提前一年实现“十五”计划，顺利完成市十三届人民代表大会历次会议确定的目标任务，荣获“国家环境保护模范城市”、“全国绿化模范城市”、“全国科技进步先进市”、“全国民族团结进步模范市”、“全国双拥模范城”、“全国最佳商业百强城市”、“中国投资环境百佳城市”等多项荣誉称号，在推进市第七次党代会确定的“率先建成全面小康社会、加速构建和谐马鞍山”征程上迈出了坚实步伐。
     （一）经济实力显著增强。全市生产总值由2002年的176亿元跃升至2007年的540亿元，五年增长2倍多，年均递增17.4%，经济总量由全省第12位上升至第4位。人均生产总值达到5600美元，继续保持全省第一，与长三角平均水平差距缩小了25%。规模以上工业增加值达到290亿元，占全省的份额由10%提高到13%，年均增长33.8%；规模以上工业企业由148户增至530户，年销售收入超亿元企业由13户增至50户。建筑业步入快速发展期，2007年产值突破100亿元，在促进农民工就业、提升综合经济实力等方面发挥了重要作用。财政收入跃上90亿元新台阶，是2002年的近4倍，年均增长30.1%。2007年全社会固定资产投资达到330亿元，五年累计1036亿元。城乡市场在规范中持续繁荣，社会消费品零售总额达到86.4亿元，年均增长14%。去年末，金融机构本外币存款余额410亿元，贷款余额303亿元，分别比2002年末增加255亿元和205亿元。在经济持续快速增长的同时，节能减排成效显著，全市万元生产总值综合能耗年均下降4.6%，重点工业企业污染源排放达标率达95%。在省政府对全省各市年度目标考核中，从2003年起，连续3年位居第一，2006年名列第二。自2003年跻身中国综合实力百强城市以来，排名由第94位前移至2006年的第74位。
     （二）结构调整迈出实质性步伐。强力推进三大经济板块、三大基地和科技创新能力建设，促进经济结构的优化调整和发展方式的加快转变。在三大经济板块中，始终把马钢摆在重中之重的位置予以支持。马钢投入400多亿元，建成了冷热轧薄板、彩涂板、“十一五”前期技术改造和结构调整系统工程等一批重大项目，成功重组合钢主业，产品结构更趋优化，总体生产规模迈上年产1600万吨钢新平台，实现销售收入五年翻两番，创造了马钢发展史上的新奇迹。举全市之力支持和推动三大省级开发区竞相发展。马鞍山经济开发区入驻企业356家，其中亿元以上内资项目20个、1000万美元以上外资项目24个，主要经济指标以年均50%以上的速度增长，综合实力连续四年位居全省省级开发区首位；慈湖??、内资53亿元，跃居全省省级开发区第4位；当涂经济开发区入驻企业90家，投资规模超过80亿元，快步进入全省省级开发区第一方阵。县区发展取得重大进展。当涂县经济总量增长近2倍，财政收入突破10亿元，连续四年位居全省经济十强县前列；花山、雨山、金家庄三个城区财政收入全面突破3亿元；三城区工业集中区初具规模，已有183个项目建成投产。目前，马钢、开发区、县区三大经济板块并驾齐驱、共同支撑全市经济发展的格局基本形成。
     围绕打造现代加工制造业基地，着力实施“万千百十”促进工程，中小企业总量突破9000户，机械制造、磁性材料等产业集群初具规模。华菱重卡底盘、星马专用汽车、山鹰纸板技改、雨润肉制品一期工程等项目相继投产，重点骨干企业核心竞争力明显增强。大力调整农业结构，加快建设绿色食品基地，全市农业实现增加值达21亿元，年均增长10.5%。农民种粮积极性得到有效保护，50亩以上粮食规模种植户近600户。养殖业占农业总产值比重达60%，水产生态养殖模式在全国推广，当涂县河蟹标准化养殖示范区通过国家验收。26个产品获得无公害农产品认证，32个产品获得绿色食品认证。农产品自营出口创汇实现零的突破，安民农产品贸易中心进入全国“双百”市场行列。加大休闲旅游基地建设力度，深度开发采石风景区旅游资源，全面拉开花山商贸旅游园建设框架，大力培育特色旅游精品，旅游业五年总收入65亿元，年均增长18.6%。出台实施服务业跨越式发展实施纲要，引进家乐福、华润苏果等国内外知名商业企业，以连锁经营、物流配送和电子商务为代表的新型流通业态不断扩大。大华国际广场、解放路和湖西南路商业街商贸服务功能得到加强，市级商业中心格局正在加速形成。
     加强自主创新能力建设。国家“863”新材料成果产业化基地建设取得重要进展，国家金属矿产资源高效循环利用工程研究中心加快建设，国家金属矿山固体废弃物处理与处置工程技术研究中心通过科技部验收。科创中心被批准为国家级创业中心，留学人员马鞍山创业园获准设立，科技创新载体和服务平台进一步完善。积极开展产学研合作，与清华大学、南京大学、北京科技大学、安徽工业大学等10多所高校签订了全面合作协议，实施产学研合作项目200多项。以企业为主体的科技创新工作扎实开展，马钢“热轧H型钢产品开发与应用技术研究”获得国家科技进步二等奖，华菱公司成为我国最具成长性的60户自主创新企业之一。质量兴市工作走在全省前列，“马钢牌”H型钢、火车车轮和“海狮牌”毛巾3个产品跻身中国名牌行列，10个产品成为国家免检产品，35个产品被评为省名牌产品，培育发展19件省著名商标。全市高新技术产业产值达到235亿元，年均增长43.2%，全市科技进步贡献率达43%。高层次人才培养、引进和使用力度加大，马钢、星马博士后科研工作站相继建立，引智项目实施水平全面提升。
     （三）改革开放实现历史性突破。按照“三个置换、两个结合、一个保障”的思路，全面完成430户国有、集体中小企业产权制度改革，对近10万名职工进行了身份置换和妥善安置，提前实现省政府确定的中小企业改革目标。全力支持大企业改革，马钢等企业办社会职能基本剥离，十七冶整体改制成为产权多元化大型施工企业，马鞍山矿山研究院成功实行企业化改制，马鞍山钢铁设计院改制成为股份制国际工程公司。完成市属3户资产运营公司重组工作，国有资产实现保值增值。新增星马汽车、中钢天源、方圆支承3户上市公司，上市公司股权分置改革如期完成。中部地区扩大增值税抵扣范围税收政策在我市顺利实施。大胆探索公用事业单位改革，城市供水、供气、公交行业改革走在全国前列。生产经营类事业单位基本完成转企改制，教育、卫生、文化等系统事业单位人事制度改革加快推进。深入实施行政管理体制改革，全面完成市政府和县区政府新一轮机构改革，成功进行新一轮对区放权和乡镇区划调整，马鞍山经济开发区、慈湖经济开发区分税制财政管理体制相继建立。机关事业单位工资套改、规范公务员津补贴工作平稳进行。交通银行马鞍山分行挂牌运营，市商业银行成功参与徽商银行联合重组，在全国组建首家市级农村合作银行。农村综合改革在当涂县完成试点的基础上，向三城区全面推开。
     深入实施大开放主战略，扎实推进东向发展。全力以赴开展招商引资，五年实际利用外资9亿美元，实际利用内资368亿元，年均分别增长52.3%和71.8%；引进世界500强企业项目及分支机构5个，促成蒙牛乳业、雨润肉制品、广州立白等国内知名企业落户，利用内外资规模和质量明显提升。三个省级开发区开放型经济载体功能持续优化，产业集聚效应不断增强。马鞍山口岸经国务院批准对外??鞍山港跻身长江港口十强行列。五年累计完成外贸进出口总额73亿美元，出口产品结构进一步优化，出口实绩企业由25户增至85户，外经营业额、外运量、境外劳务输出人员数量成倍增长，全市经济外向度由17.6%提高到31.9%。外事、侨务、外宣工作全面展开，新增国际友好城市2个，取得一定程度的外事审批权，对外交流合作不断拓宽。区域合作进一步扩大，融入长三角和共建南京都市圈、皖江城市群工作取得积极成果。
     （四）城市综合服务功能日趋完善。按照建成区100平方公里的目标，对城市总体规划进行修编，相继完成了59项重大城市规划编制工作。以完善城市路网和公用设施为重点，大规模展开城市建设，建成区面积由40平方公里扩大到近70平方公里，城市化率由51%提高到61%。城市南部和东部路网等一批主干道先后建成，新增市区道路总里程221公里。建成湖西南路，开辟了市区连接当涂县城的新通道。马芜高速公路建成通车，马鞍山长江公路大桥实施保障工程开工建设，城市对外交通体系日趋健全。一批自来水、污水处理、生活垃圾处理、天然气工程相继建成运行，供电、邮政、通讯设施不断完善。房地产市场健康发展，五年累计竣工住房面积600多万平方米，实现城镇人均住房面积新增9平方米，达到29.8平方米。住房公积金归集额、贷款发放额年均分别增长29.4%和57.9%，当涂县公积金管理与市区接轨。生态市建设成效显著。全面实施了雨山湖综合整治，建成市政公园并对外开放，完成了佳山、雨山公园规划建设，新建了采石河节庆广场等一批主题公园和市民广场，市区绿化覆盖率提高到43%。一批重点污染源得到集中整治，城市环境空气质量优良率达90%以上。当涂县城建设日新月异，作为城市副中心的构架初步拉开。小城镇建设累计完成投资36.3亿元，中心镇服务功能明显增强。依法加强城市管理，集中开展城市综合整治，违法建设拆除、散装货物车辆密闭改装、“四小车辆”专项整治、城市美化亮化等工作取得明显成效，市容市貌进一步改观。继续规范相对集中行政处罚权制度，推行市容环卫领域网格化管理，市容环境卫生管理逐步长效化。
     （五）新农村建设稳步推进。坚持统筹城乡发展，先后出台落实支持“三农”的政策措施近90项，在全省率先免征农业税及附加，五年各级财政投入“三农”资金近20亿元。编制了新农村建设总体规划、产业发展规划、村庄布点规划和各专项配套规划。当涂县被确定为中央财政整合资金支持新农村建设试点，全市有四镇二十村被列为省新农村建设“千村百镇”示范点，三杨、丹东等示范点建设取得新成绩。大力发展现代农业，新增省级农业产业化龙头企业5户，其中2户营销收入超5亿元；累计引进建设各类“三资”农业项目691个，到位资金30亿元；新建农民专业合作社38家；苗木花卉基地建成面积近万亩，速生丰产林基地发展到2.6万亩，奶牛养殖基地养殖奶牛近万头，无公害蔬菜基地发展到2.1万亩。不断加大农村基础设施投入。新建改建农村道路1300余公里，农村交通条件进一步改善。“村村通水泥路”工程和“农村安全饮水”工程在全省率先完成，农村电网建设改造一期、二期工程完工，列入国家重点投资项目的江心乡万亩土地整理项目获得批准。病险水库除险加固顺利完成，病险涵闸除险改造加快实施。基本农田保护措施得到加强，土地征迁和农民安置工作有序开展。加速推进“万村千乡市场工程”，启动“新网工程”建设，农村消费品市场日趋健全。加快发展农村社会事业，当涂县中小学D类危房全面改造，乡镇卫生院医疗条件得到改善，6个省级农村文化站示范点启动建设，20户以上自然村“村村通有线电视”工程稳步实施。
     （六）社会建设跃上新台阶。教育现代化步伐加快。素质教育深入实施，“普九”成果得到巩固，义务教育阶段“两免一补”政策全面落实。二中新校区等一批学校建成招生，中小学办学条件普遍改善，基础教育向均衡方向积极推进。普及高中阶段教育全面提速，3所中学成为省示范高中，高中阶段教育毛入学率达到73%，实现较大提高。职业教育快速发展，马鞍山技师学院建成招生，安工大职业技术学院一期工程、当涂县职教中心相继竣工，全市高中阶段职普招生比接近1:1。高等教育规模快速扩张，马鞍山师范学校升格为师范高等专科学校，建成马鞍山网络大学，安工大新校区一期工程投入使用，引进河海大学创办独立学院取得实质性进展。学习型城市建设向纵深推进，花山区被评为“全国家庭教育示范区”。文化事业日趋繁荣。举办了建市50周年庆典、第一届中国诗歌节、国际太极拳邀请赛、中央电视台陈式太极拳全国选拔赛等重大活动，专业艺术创作演出成果丰硕。基层文化活动丰富多彩，花山区东苑社区和金家庄区杨桥社区被评为“全国先进文化社区”，金家庄区映山红艺术团赴港参赛荣获“金紫荆花”大奖。李白墓园成为国家重点文物保护单位，当涂民歌进入国家非物质文化遗产名录。博物馆、大剧院、新图书馆加快建设，基层文化基础设施进一步完善。公共卫生服务体系逐步健全。先后建成市疾控中心、市紧急救援中心和市传染病医院一期工程，重大疾病预防控制工作得到加强。雨山区成为全省首个“全国社区卫生服务示范区”。医疗卫生服务水平有了新提高，全市人口平均预期寿命达78.8岁。成功举办市第九届运动会，竞技体育迈上新台阶，群众性体育活动蓬勃开展，金家庄区新风社区荣获“全国群众体育先进社区”称号。低生育水平持续稳定，全市年均人口出生率控制在10.5‰以内，出生人口政策符合率保持在95%以上，农村部分计划生育家庭奖励扶助制度全面实施。精神文明建设深入开展，顺利通过国家卫生城市复核检查和安徽省文明城市考核验收，文明创建工作经验被中央媒体先后两次集中报道。信用马鞍山建设迈出新步伐，5户企业被评为国家级“守合同重信用”单位。高质量完成第一次经济普查和第二次农业普查。和谐社区建设扎实开展，农村社区建设试点成效明显，基层民主政治建设得到加强。社会科学进一步繁荣。雨山区被命名为“全国科普示范城区”。《马鞍山市志》续志工作基本完成。档案管理模式改革试点积极推进。对台经贸、文化交流不断增多。接待、地震、地质工作卓有成效，民族、宗教工作水平不断提高，妇女、儿童、老年人事业取得新进展。
     大力创建平安马鞍山。严密防范、严厉打击各类违法犯罪活动，积极构筑社会治安防控体系，“平安技防”工程初步建成，社区矫正工作全面推开，社会治安综合治理工作成效显著，人民群众对治安状况的满意率达97.8%。雨山区被评为“全国社会治安综合治理先进集体”，花山区跻身首批省级“平安区”行列。法制宣传、依法治市工作深入开展，法律援助扎实推进，荣获“全国四五普法先进城市”称号。仲裁工作在解决经济纠纷方面做出了积极贡献。实施领导包案、开门接访和带案下访等制度，去省进京上访量逐年下降，成为全省信访形势最好的城市之一。认真落实安全生产责任制，强化非煤矿山等安全专项整治，加大重大安全隐患排查治理力度，安全生产形势总体平稳。在全国地级市中率先建成新一代天气雷达站。全市突发公共事件应急预案体系基本建成，防范和应对公共危机能力明显增强。国家安全工作卓有成效。人民防空事业受到国家国动委表彰。积极支持驻马部队建设，国防指挥中心、武警支队机关营房、消防支队特勤消防站建成使用；海军“马鞍山舰”正式命名；连续五届荣获“省双拥模范城”称号。
     （七）人民生活水平大幅提升。城市居民人均可支配收入达16100元，农民人均纯收入达6100元，均比2002年增长1倍以上，年均分别增长15.8%和15.9%。城乡居民人均储蓄存款达到16355元，年均增长16%，居民财富积累显著增加。扎实推进创业富民和就业富民工程，出台落实30项政策措施，降低创业门槛，培育创业主体，支持工会、共青团、妇联开展结对帮扶、青年创业、巾帼创业等援助活动，全民创业环境不断优化。五年累计新增私营企业5418户，民营经济增加值年均增长25%，对全市经济增长的贡献率达40%以上。完善积极的就业再就业政策措施，广泛开辟就业渠道，实行“一对一”职业介绍服务，统筹推进城乡劳动者就业。五年累计新增就业14.1万人，其中下岗失业人员再就业8.7万人，“4050”人员等困难群体再就业1.5万人，城镇登记失业率由4.2%下降至3.3%，被国务院授予“全国就业再就业工作先进单位”。在全国首创村级劳务公司，全市所有乡镇、村均建立了劳动保障工作机构，建成70个充分就业社区，年均在外务工经商人员达21.3万人。建立全省首个“大学生创业园”，高校毕业生就业促进工作取得新突破。实施下岗失业人员再就业、农村劳动力转移就业和创业三大培训工程，五年累计培训7.6万人，高技能劳动者比例达到19%。稳步推进劳动合同和集体合同制度，有效解决了涉及农民工利益问题。不断发展和谐劳动关系，马钢、马鞍山经济开发区分dquo;和谐工业园区”称号。
     不断健全城乡社会保障制度。继续加大城镇养老、失业、医疗保险扩面力度，覆盖率均达98%以上。建成城镇职工工伤、生育保险制度，参保人员分别突破20万人和16万人。企业退休人员管理服务全面实现社会化。在全省率先实施被征地农民基本养老保障制度，实现即征即保。率先建成城镇居民基本医疗保险制度，全面推行新型农村合作医疗制度，实现基本医疗保险城乡全覆盖。率先建立新型农村社会养老保险制度，农民养老方式向社会化迈出重要步伐。社会救助体系框架基本建成。城市低保工作实现应保尽保、分类施保、动态管理，建立农村低保制度，率先实现低保制度城乡全覆盖。实施城乡贫困群众医疗救助制度、低保对象重病和农村特困户、五保户大病救助，农村敬老院改扩建工作力度加大。慈善事业持续健康发展，荣获“中华慈善事业突出贡献奖”。残疾人就业权利得到切实维护，雨山区被评为“全国残疾人工作示范区”。落实廉租住房制度和低收入家庭住房补贴制度，实施城市危旧房改造50万平方米，建成2万多套被征地农民安置房，基本消除农村人居草房。全面提高企业退休人员养老金、失业人员失业保险金和劳动者最低工资标准、城乡低保标准、五保供养标准，低收入者收入水平稳步提升。前四年，市政府共实施40件为民办实事项目，解决了群众关注的一批热点、难点问题。雨山湖公园等城市公园免费向市民开放，累计改造背街小巷道路117条、老旧小区15个。2007年，全面实施30项民生工程，其中省政府确定的12项民生工程任务率先完成。
     （八）政府自身建设全面加强。五年来，市政府向人大报告工作、向政协通报情况制度得到全面落实，政府系统承办的41件人大代表议案、490件建议批评意见、16件市人大常委会审议意见、9件市人大主任会议意见、1700件政协提案，均按要求办复。制定实施市政府负责同志领衔办理人大代表议案、政协提案制度，市政府班子成员定期约请和走访、看望市人大代表、市政协委员，市政府部门积极加强与各民主党派、工商联和人民团体对口联系，听取对政府工作的意见和建议。
     全市政府系统认真开展保持共产党员先进性教育活动，踊跃参与以“东向发展、全民创业”为主题的新一轮解放思想大讨论，取得了促进各项工作的明显成效。建立了市政府学习扩大会制度，各级行政领导综合素质和施政水平有了新提高。完善重大决策的规则和程序，广泛集中民智，促进了决策的科学化、民主化。出台落实《市政府工作规则》及具体规定，制定实施提高行政领导工作效能、行政效能监察相关制度，实行政府部门行政首长问责制，推行行政效能建设六项工作制度，政府系统效能建设不断加强。取消和调整600余项审批事项，对部分审批和公共服务流程进行再造，推行授权委托审批；减少行政性收费项目300余项，由市政府埋单、暂免9个部门37项行政事业性收费，行政行为得到规范，对公共行业的监管更加有力。加大重大决策事项督查力度，不断改进目标管理办法，激励和鞭策作用明显增强。在全省率先推行现行文件公开查阅制度，实行市政府公报免费直送制度，政务公开深入推进。扎实开展机关作风和效能评议，认真办好市长公开电话和“政风行风热线”，政风行风建设连续五年全省考核“优秀”。强化审计监督，实行政府投资超百万元建设项目“全覆盖”、“必审制”，首次向社会公开本级预算执行审计结果。切实开展政府投资和重点项目专项执法监察，深入推进商业贿赂专项治理，加大违法违纪案件和问题的查处力度，及时纠正损害群众利益的不正之风，廉政建设得到进一步加强。
     各位代表！2007年是市第十三届人民政府任期最后一年。一年来，市政府坚持东向发展和全民创业，加快实施重点项目建设，大力推动产业结构优化升级，全面发展各项社会事业，着力提高人民生活水平，保持和扩大了又好又快的发展势头。预计全市生产总值比上年增长17.2%；财政收入增长18%；固定资产投资增长25.8%；实际利用外资3.5亿美元，实际利用内资148亿元，分别增长71%和33%；社会消费品零售总额增长17.6%，是1995年以来的最高增幅；实现进出口总额23亿美元，其中出口8亿美元，增长46.8%；城市居民人均可支配收入增长17.4%；农民人均纯收入增长17.5%；万元生产总值综合能耗下降4.5%，超额完成省政府下达我市的污染减排任务。2007年全市经济社会发展取得显著成绩，全面超额完成了本届政府各项目标任务。
     五年的政府工作实践，使我们深刻体会到，必须坚持以邓小平理论和“三个代表”重要思想为指导，深入学习贯彻科学发展观，以先进的理念引导发展实践，不断拓宽发展思路，创新发展举措，努力走出一条符合时代要求、具有自身特点的发展之路。必须坚持发展第一要务，牢牢扭住经济建设这个中心，坚持不懈地走新型工业化道路，抢抓每一次发展机遇，做到早谋划、早启动，以重点突破带动全局，不断开创经济社会又好又快发展新局面。必须坚持深化改革、扩大开放，坚定不移地推动重要领域和关键环节的改革，积极实施大开放主战略，用改革、开放和发展的办法解决前进中的困难和问题，不断激发经济社会发展的动力和活力。必须坚持全面协调可持续发展，按照生产发展、生活富裕、生态良好的要求，加快建设资源节约型、环境友好型社会，实现经济社会永续发展。必须坚持以人为本、高度关注民生，按照共建共享的要求，把实现好、维护好、发展好最广大人民群众的根本利益作为政府一切工作的出发点和落脚点，让改革发展成果最大程度地惠及广大人民群众。必须坚持依法行政，加快推进政府职能转变，改进优化工作流程，不断提高行政效能，为经济社会发展、人民安居乐业创造良好的政务环境。
     各位代表!过去五年，是全市综合实力不断跃升、城乡面貌显著变化的五年，是改革开放取得突破性进展、经济社会发展活力明显增强的五年，是和谐社会建设加速推进、人民群众得到实惠更多的五年。成绩来之不易，这是省委、省政府和市委正确领导的结果，是全市上下、方方面面团结奋斗的结果。在此，我代表市人民政府，向辛勤工作在全市各条战线上的广大人民群众，向长期以来给予政府工作支持和帮助的人大代表、政协委员和各民主党派、工商联、人民团体及社会各界人士，向驻马解放军、武警部队官兵和全市政法干警，向中央、省驻马单位和在我市创业的境内外投资者、建设者，表示衷心的感谢、致以崇高的敬意！
     我们也清醒地看到，当前我市经济社会发展中还有不少矛盾和问题，政府工作也存在一些差距和不足。突出表现在：一是与长三角发达城市比，人均经济指标仍存在较大差距，追赶的任务非常艰巨；与省内先进城市比，发展步伐需要进一步加快。二是经济增长的环境、资源、成本约束日趋严重，人才支撑不足，转变经济发展方式尤为迫切。三是随着社会转型步伐加快，社会建设和管理面临着一定的难度和复杂性，公共服务均等化水平有待提高；少数群众生活还比较困难，统筹各方面利益的长效机制需要进一步健全，构建和谐马鞍山任重道远。四是政府职能转变还不能完全适应新形势新任务的要求，政府一些工作人员素质不高、服务不到位的现象还一定程度存在，发展的软环境仍需进一步改善。对此，我们一定要认真对待，在今后的工作中努力加以解决。
    二、今后五年的奋斗目标和主要任务
     今后五年，是马鞍山深入贯彻党的十七大精神、率先建成全面小康社会、并向率先基本实现现代化迈进的关键时期。纵观全局，我们面临着十分难得的战略机遇。经济全球化在新的广度和深度上继续拓展，特别是2008年奥运会、2010年世博会相继在我国举行，为我市扩大对外开放提供了广阔空间。随着国家中部崛起战略和省东向发展战略的全面实施，我市在区域发展格局中的地位必将进一步提升。经过多年的建设和积累，我市已进入人均生产总值从5000多美元向更高水平跃升的新阶段，发展的能量更大，发展的活力更强。在这样一个重要阶段，抓住机遇，就能实现更大规模、更高水平的发展和跨越。我们一定要以高度的历史使命感、强烈的忧患意识和宽广的时代眼光，紧紧抓住重要战略机遇期，积极应对各种挑战，励精图治，锐意进取，敢有作为，善有作为，在新的起点上再攀新高峰。
     今后五年政府工作的指导思想是：全面贯彻党的十七大精神，高举中国特色社会主义伟大旗帜，以邓小平理论和“三个代表”重要思想为指导，深入贯彻落实科学发展观，牢固坚持发展第一要务和经济建设这个中心不动摇，以实施工业强市、促进协调发展为主题，以转变发展方式、优化经济结构为主线，以推进东向发展、深化改革创新为动力，以促进社会稳定和谐、提高人民生活质量为根本，全面推进经济、政治、文化、社会建设，率先建成更高水平的全面小康社会，不失时机地朝着率先基本实现现代化迈进。
     今后五年总的奋斗目标是：又好又快新跨越，率先全面达小康。在具体工作中，要通过三步走，实现“位次再前移，总量再翻番，水平再提升”，即：在优化结构、提高效益、降低消耗、保护环境的基础上，到2010年，全面完成“十一五”规划，努力提升经济总量在全省的位次，努力提升人均经?生产总值翻两番；到2011年，在全省率先建成经济发展更具协调性、社会主义民主进一步扩大、文化建设明显加强、社会事业加快发展、生态文明显著提升，符合党的十七大要求、具有更高水平的全面小康社会；到2012年，生产总值迈上1000亿元新台阶，主要总量指标比2007年翻一番，成为带动皖江城市群加速跨越的龙头、促进安徽奋力崛起的先锋、中部地区具有较强影响力的城市。
     今后五年政府的主要任务是：
     ——加快发展具有鲜明特色和较强竞争力的现代产业体系。坚持走新型工业化道路，立足现有产业特点，促进信息化与工业化融合，做大做强第二产业，提升壮大第三产业，调整优化第一产业。到2012年，形成以马钢为龙头的销售收入千亿级的钢铁产业和百亿级的汽车制造、食品加工、磁性材料等企业集群，打造一批全国领先“单打冠军型”企业，规模以上工业企业达1200户，规模以上工业增加值超700亿元、销售收入突破2000亿元；服务业实现跨越式发展，增加值五年翻一番；大力发展规模特色农业，形成高效农业快速发展新格局。
     ——进一步提升外向型经济发展水平。强力实施东向发展战略，合力推进皖江城市群和南京都市圈建设，加速融入长三角，积极参与国际国内经济大循环。五年实际利用外资超20亿美元，实际利用内资超1000亿元，更多地引进国内外先进技术、管理经验和高素质人才。力争马鞍山经济开发区升格为国家级开发区，博望工业园成为首批国家级特色产业园。五年实现进出口总额160亿美元以上，外贸货运量年均增长20%以上，对外经济合作规模明显扩大。
     ——深入推进经济社会等领域体制改革。坚持和完善基本经济制度，建立健全现代企业制度，推动企业体制机制创新。加快建设现代市场体系，积极发展各类要素市场和区域性专业市场，大力发展各类市场中介组织。鼓励、支持、引导非公有制经济发展，扎实推进全民创业，保持私营企业以每年1500户以上的速度递增，实现中小企业总数超1.6万户。按照加大力度、加快进度、取得实质性进展的要求，推动文化体制改革在面上展开、向纵深推进。实行政事分开、管办分开、医药分开、营利性和非营利性分开，加快医疗卫生事业改革和发展步伐。分类推进行政执行类、社会公益类事业单位改革，进一步深化事业单位人事制度改革。加快行政管理体制改革，着力转变职能、理顺关系、优化结构、提高效能。积极推进乡镇区划调整，进一步激发重点镇发展活力。
     ——扎实做好自主创新和节能减排工作。把提高自主创新能力作为转变经济发展方式的中心环节，基本建立起以企业为主体、市场为导向、产学研相结合的技术创新体系，引导和支持创新要素向企业集聚，促进科技成果向现实生产力转化，建设特色鲜明的创新型城市。到2012年，科技进步贡献率达50%以上，全社会研究开发经费支出占生产总值比重达2%以上，高新技术产业增加值占工业增加值比重达30%以上。大力实施人才强市和知识产权战略，优化人才资源结构，加强高层次人才引进和培养，把我市建成创新创业人才竞显才华的基地。把节能减排作为转变经济发展方式最现实有效的突破口，引导经济社会发展转入科学发展的轨道，基本形成节约能源资源和保护生态环境的产业结构、增长方式、消费模式。到2012年，万元生产总值综合能耗比2007年下降20%左右，主要污染物排放总量下降8%，生态文明建设保持全国同类城市先进水平。
     ——加速形成城乡一体化发展新格局。围绕“双百”现代化城市和新农村建设，全面提高城乡规划建设水平，加快中心城区能级和品位提升，促进主副城区加速对接，着力优化卫星城、重点镇功能。精心打造沿雨山湖、采石河、慈湖河生态景观带，整合开发采石地区山水资源，着力放大滨江山水园林城市特色，建成中部地区最适宜人居的城市之一。充分发挥长江公路大桥、宁宜城际铁路、芜申运河相继建成后的综合优势，全面推进与周边城市的融合，使马鞍山成为安徽接轨长三角的战略节点城市、东向发展的重要区域性资源配置中心。健全以工促农、以城带乡长效机制，积极探索统筹城乡综合配套改革试点工作，推动城市基础设施向农村延伸、城市公共服务向农村覆盖，全面提升城乡一体化发展水平。加快农村人口向城镇转移步伐，到2012年，城市化率达到70%以上。加大引导扶持力度，着力形成特色争先、整体推进的县区经济发展格局，力争当涂进入全国县域经济社会发展第一方阵，三城区和博望等重点镇经济发展水平走在全省前列。
     ——全方位加强社会建设。坚持富民优先，通过支持创业增加经营性收入，依靠扩大就业增加工资性收入，积极创造条件增加财产性收入，合理调整分配逐步扭转收入差距扩大趋势。以统筹城乡就业为主线，以鼓励创业带动就业，实现城乡劳动者充分就业。加大投入力度，推动公益性文化事业加快发展。大力发展经营性文化产业，到2012年，实现文化产业增加值占生产总值比重达到4.5%。深入开展群众性精神文明创建活动，力争早日进入全国文明城市行列。优先发展教育事业，均衡发展义务教育，基本普及高中阶段教育，推进职业教育集约化发展，力争高等教育毛入学率达到30%以上。加快建设学习型城市，构建终身教育体系。完善全民健身活动场所，增强城乡居民健康体能素质。建立事业单位养老保险制度、城镇居民养老保险制度和覆盖城乡居民的基本医疗服务体系，健全住房保障制度，完善全覆盖、多层次、较高水平的社会保障体系。高度重视和关心城市外来人口的生产生活，引导他们逐步融入城市社会。进一步加强社会治安综合治理和信访、安全生产工作，不断提高突发事件应急保障能力，形成促进稳定和谐的社会管理新格局，力争进入全省首批平安市行列。
     ——努力建设人民满意政府。建立公开透明、程序规范的行政决策、执行和监督机制，完善决策信息和智力支持系统，促进政府决策更加科学、民主。全面推进依法行政，加快建设法治政府。大力推进政务公开，努力建设“阳光政府”。加强基层政权建设，发展基层民主，实现政府行政管理与基层群众自治有效衔接和良性互动。进一步完善目标管理考核机制，推进机关作风和效能建设，优化经济社会发展环境。全面贯彻《公务员法》及相关配套法规，努力建设一支政治坚定、业务精湛、作风过硬、人民满意的公务员队伍。大力倡导和践行“八个方面良好风气”，完善以规范权力运行为核心的体制机制，在政府系统形成风清气正的良好局面。
    三、扎实做好2008年各项工作
     2008年是全面贯彻落实党的十七大精神的第一年，也是新一届政府开局之年。根据中央关于“控总量、稳物价、调结构、促平衡”的经济工作方针，综合考虑多方面因素，确定今年全市经济社会发展的主要目标为：生产总值增长13%以上；财政收入与生产总值同步增长，突破100亿元；全社会固定资产投资增长20%；社会消费品零售总额增长14%；城市居民人均可支配收入、农民人均纯收入均增长10%以上；万元生产总值综合能耗下降4%左右，主要污染物排放量下降2%；城镇登记失业率控制在4%以内；居民消费价格涨幅和人口自然增长率控制在省下达的指标范围内。为此，我们将重点做好以下九个方面工作。
     （一）着力做强做优工业经济。认真贯彻全省工业强省大会精神，加快推进产业结构升级和自主创新，大力实施品牌战略，不断增强工业经济综合实力和竞争力。
     全力推动马钢更好更快发展。支持马钢新区实现顺产稳产，促进马钢全年实现销售收入超600亿元。积极配合马钢做好实施“十一五”后期技术改造和结构调整补充规划的各项准备工作，建成高速车轮适应性改造和大型锻钢工程，加快推进硅钢生产线项目建设，开工兴建轻苯精制改造工程，不断增强发展后劲。继续支持马钢推进和尚桥、白象山等后备矿山建设，为马钢更大发展提供资源保障。支持马钢加快生产指挥中心建设，推动管理创新再上新台阶。
     大力实施工业“千百十”行动计划。继续以项目建设为抓手，集中力量实施一批重点工业项目。积极支持华菱公司实施5万辆重卡规划，推动与世界著名大企业合资合作。加速推进山鹰瓦楞纸板技改、大唐燃煤发电一期工程、蒙牛三期常温液态奶生产线、立白日用化工产品生产、天成纺织搬迁等一批重点项目建设进度，开工建设马鞍山发电厂两台60万千瓦机组改扩建工程，加大广东科达机电项目前期工作力度。支持县区加快红太阳吡啶、长江钢铁技改扩能、浙江工业园、迪昂风能发电设备、阳晨生物蛋白等项目建设步伐。全面落实促进中小企业发展各项政策措施，加快培育一批高成长性中小企业，全年新增规模以上工业企业100户，中小企业总量突破1万户。扎实实施产业集群规划，建设汽车零部件、钢铁深加工等产业集群，培育和菱、中钢天源等集群龙头企业，推动博望机械刃模具产业集群装备和产品升级，规划建设沿江船舶产业园。深入实施质量兴市和名牌战略，组织开展企业质量振兴活动，鼓励企业争创名牌产品和驰名商标。力争全年工业企业销售以上名牌产品10个。
     切实增强企业自主创新能力。多渠道加大科技研发投入，加强骨干企业及科技型中小企业创新能力建设，培育一批省、市创新型试点企业。全面建成国家“863”新材料成果产业化基地主体园区，发挥新材料基地对高新技术产业的示范带动作用，高新技术产业增加值占工业增加值的比重提高2个百分点。积极申报马鞍山经济开发区成为省级高新技术产业开发区，推进花山软件园、向山电子元件及磁性材料园、博望国家星火技术密集区建设。启动建设国家级钢铁及制品质量监督检验中心，新增市级技术中心10个以上。
     （二）掀起改革开放和全民创业新热潮。今年是改革开放30周年。我们要通过继续深化改革、进一步提高对外开放水平、不断扩大全民创业成果，努力在完善体制机制和激发经济发展活力上取得新突破。
     在更高层次上推进对外开放。积极创新招商方式，合理配置驻点招商力量，加强专业化招商队伍建设，精心组织开展系列招商活动，不断增强招商引资的针对性、有效性。突出重点区域，以引进国际国内大集团、大企业和大项目为重点，形成重大项目在建、在谈、储备良性循环。拓宽招商领域，实行产业链招商，引导外资加快发展汽车零部件、船舶等先进制造业和软件开发、物流配送等现代服务业。建立健全招商引资项目信息共享、落地服务、协调推进机制，努力提升招商引资的层次和水平。力争全年实际利用外资4亿美元，实际利用内资180亿元。全力推进马鞍山经济开发区南区建设，完成“一纵三横”道路网，配套完善供电、排水、排污等市政设施。进一步完善慈湖经济开发区大项目承载功能，助推产业升级，逐步实现绿色转型。支持当涂经济开发区功能开发，扶持三个城区的各类园区加快升级步伐。推动外经贸加快发展。进一步提高机电、高新技术产品出口比重，扩大关键设备、技术和重要资源性产品进口，全年完成外贸进出口总额22亿美元。支持中冶华天、十七冶等大企业对外承揽技术含量高、带动成套设备、技术出口和劳务输出强的大型工程总承包项目，推动已签约工程项目尽快实施，加快边防检查站建设和外贸码头改造步伐，力争全年外经营业额、境外劳务输出人数比上年增长20%。进一步深化与长三角地区的合作对接，全方位参与南京都市圈建设，大力拓展与皖江城市的合作领域，更好地担当全省东向发展排头兵的重任。
     加大企事业单位改革力度。巩固和完善企业改革成果。支持马钢实施整体改制，实现集团公司从资产经营型向资本运营型的转变。加强优势企业上市资源培育，力争2户企业上市。整合各类担保公司，完善小企业贷款风险补偿和奖励办法，努力缓解融资困难。健全国有资产经营业绩考核，全面加强企业风险管理，提高国有资产监管水平。启动事业单位机构改革工作，深化事业单位人事、收入分配制度改革。落实国家金融改革各项措施，完善银行业体系建设，支持金融机构向农村布点。
     深入推进全民创业。进一步完善和落实促进全民创业的政策措施，形成有利于各类主体竞相创业的体制机制。引导社会资源参与创业服务体系建设，大力发展辅导孵化、管理咨询、人才培训等服务组织和中介机构。完善县区创业辅导机构功能，探索建立乡镇、街道创业辅导机构，广泛开展全程帮扶行动。加强创业载体建设，建成创业基地10万平方米。在丰富创业内涵、提升创业档次的前提下，新增私营企业1500户以上。
     （三）全面发展以生产性服务业为重点的第三产业。大力实施服务业跨越式发展实施纲要，整体提升服务业发展水平。
     加速发展物流业。充分发挥区位和港口优势，全面实施《马鞍山市物流业发展规划》，全年物流总量突破1亿吨。整合长江岸线资源，推进人头矶码头、天顺码头和长江港口码头建设，新建慈湖经济开发区华东物流园，扶持和培育港口集团、长运、联运、快捷物流等第三方物流企业做大做强。建立市级物流公共信息平台，实现物流供求信息资源共享。以南京区域现代物流联盟为纽带，加强区域物流合作。
     培育壮大新兴服务业。在冶金技术、工程设计、加工制造等优势领域发展新兴外包服务，促进会展业加快发展。加大信息网络建设投入，开展社区信息化试点，提升中小企业信息化水平，推动农村信息化重点项目建设。创新金融保险服务产品，积极发展金融保险业，促进经济金融良性互动。进一步提升社会化养老、家政、社区服务业档次，加快发展律师、公证、咨询、会计等中介服务业，不断提高服务业整体发展水平。
     改造提升商贸旅游业。推进大华国际广场二期、沃尔玛超市等重点项目建设，加大解放路、湖东路、湖南路等传统商业街的改造升级，增强中心商贸区集聚效应。提升花山路、红旗中路建材街档次，改造采石旅游购物街，新建红东餐饮街和新城东区商贸园，打造湖东路电子产品街和雨山西路商贸区，促进特色街区功能完善。全面启动第二批小商品市场建设，建成安民农产品贸易中心二期工程。完善“万村千乡市场工程”，加快推进“新网工程”建设，进一步健全城乡生活和生产资料营销网络。巩固“旅游发展年”活动成果，大力整合旅游资源，推动花山商贸旅游园项目建设，下功夫开发乡村生态旅游，建成海外海五星级酒店，实现全年旅游业总收入增长30%以上。
     （四）加快农村经济社会发展步伐。以农民增收为核心，以发展现代农业、繁荣农村经济为首要任务，充分发挥农民主体作用，努力开创新农村建设新局面。
     大力发展高效特色农业。扩大无公害蔬菜、苗木花卉基地面积，筹建东部花木市场；加快当涂河蟹出口基地建设，培植水产养殖业集散地；积极发展“一村一品”，壮大农业规模板块经济。实施“125强龙工程”，培育年营销收入超20亿元企业1户、超10亿元企业2户、超亿元企业5户，提升农业产业化水平。加快发展农民专业合作组织，完善内部管理制度，规范利益分配机制。大力发展“三资”农业，力争引进农业项目资金突破4亿元。推进农业标准化建设，新增无公害农产品、绿色食品、安徽名牌农产品10个。实施农产品市场准入制度，加强重大动植物疫病防控，确保农产品质量和安全。
     全面深化农村改革。深入推进农村综合改革，增强村级集体经济实力，创新为农服务方式，有效化解村级债务。申报全省城乡统筹发展综合试验区，适时开展统筹城乡综合配套改革试点。完善土地流转程序，推动耕地向规模经营集中。改革基层农技推广机构，推动农业科技特派员工作，逐步形成多元化基层农业技术推广体系。全面完成集体林权制度改革任务，加快农村小型水利工程产权制度改革步伐。推进供销合作社开放办社，进一步提高经营活力和市场竞争力。
     加速发展农村各项事业。推进农村中小学、乡镇卫生院、村卫生室标准化建设，扎实开展农村基层创建活动，不断提高农民综合素质。完成大公圩泵站技改工程建设，新建一五圩排涝站，完成涵闸除险改造年度任务。加强对“四镇二十村”新农村建设试点工作分类指导，落实发展措施，建立健全动态管理和领导定点联系、部门结对帮扶、村企共建制度，切实发挥示范带动作用。
     （五）继续加大城乡规划建设管理力度。坚持规划、建设、管理并重，实现外延扩张与内涵提升相结合，努力塑造城乡新形象。
     坚持规划龙头作用。贯彻实施《城乡规划法》，统一城乡规划管理。以城市总体规划为指导，科学编制城际铁路站场、长江公路大桥立交口等控制性规划，进一步建立和完善城乡规划编制体系。完善规划公示制度，强化规划实施情况监督管理，开展建设项目规划全过程监督检查，维护规划的严肃性和权威性。组织开展第二次全国土地调查，做好新一轮土地利用总体规划修编工作，完善土地征迁政策。
     加强基础设施建设。推进城市南部路网建设，拉开东部新城区路网框架，打通北部慈湖地区物流通道，改善西部地区临江道路条件。完善城市中心地区路况，加快区县二、三级道路改造升级步伐。强力推进马鞍山长江公路大桥建设，实施东环路高速化改造。贯通澄湖路、新黄路，改建通乡油路40公里，实现乡镇半小时上高速的目标。全面开展城市防洪工程建设，启动慈湖河综合整治工程，加强水系治理，逐步完善城乡排涝体系。开工建设城市东部污水处理厂、慈湖污水处理厂，加强城市供水、供气、公交设施和电网建设。加快推进市政公园后续工程，建设湖北路滨湖休闲游园等公园，提升雨山路、湖西南路等主干道路绿化水平。继续实施主干道旧房出新工程，综合整治一批老旧小区，分步实施金家庄部分地区居民整体搬迁安置，做好集体土地上房屋权属登记发证工作。完成小城镇建设投资11.5亿元，促进重点镇加快发展。调整住房供应结构，加强和改善房地产市场管理，全年竣工住宅面积110万平方米，完成危旧房改造20万平方米，着力解决城市低收入家庭住房困难。
     健全管理长效机制。继续加强城市综合整治，加大对违法建设、出店占道经营、违章户外广告、渣土抛洒、乱涂乱画等行为整治力度。完善市容环境卫生??乡结合部的环境卫生治理。启动向山生活垃圾处置场无害化处理二期工程建设，提高农村生活垃圾无害化处理率。积极推行环卫标准化管理，加大环卫作业市场化改革力度。进一步完善网格化管理，推进城市管理信息化，逐步实现城市管理工作向数字化过渡。坚持节约集约利用土地，切实加强耕地保护，不断提高单位面积投资强度和产出效益。
     （六）强力推进节能减排和生态保护工作。节约资源、保护环境事关人民群众切身利益和子孙后代生存发展，我们要以高度负责的态度，花更大气力做好这项工作。
     加大节能减排攻坚力度。认真落实节能减排目标责任制，建立健全重点行业、企业节能减排评价考核机制，确保实现年度节能减排目标。支持马钢实施高炉鼓风脱湿系统优化改造等重点节能项目建设，引导列入国家、省节能行动计划的企业加大技术改造力度，积极推广应用节能先进技术。支持万能达老机组、马钢烧结脱硫改造，加大对落后的小钢铁、小冶炼企业综合整治力度，对高耗能企业实行差别电价政策。加强节能环保监督，及时查处和整治违法违规行为。做好企业环境管理体系认证工作，推动企业开展清洁生产。
     切实提高能源资源利用效率。落实循环经济发展规划，推进慈湖经济开发区循环经济示范园区建设，实施一批循环经济示范项目。支持马钢争创“国家环境友好型企业”，加快实现余热余能全回收、工业和生活用水全循环、固体废弃物全利用。大力开发和推广节约、替代、循环利用的先进适用技术，引导扶持企业和社会利用可再生能源。建设小型沼气示范工程，促进农村循环经济发展。
     继续改善城乡环境质量。严格执行环境保护“三同时”制度，实行环境污染源头控制。深入开展环保专项行动，加强城区餐饮业油烟污染、金属加工噪音扰民和企业排污综合整治。启动危险废物集中处置中心项目建设，完成市环境监控中心建设主体工程，保障环境安全。巩固和扩大创建全国绿化模范城市成果，大力推进城乡绿化一体化，深入开展各类基层生态创建活动，加快矿区生态系统恢复和生态复垦步伐，努力建设生态文明城市。
     （七）大力繁荣发展先进文化。把今年作为“文化发展年”，兴起文化建设新高潮，推动全市文化大发展、大繁荣。
     加强精神文明建设。扎实推进社会主义核心价值体系建设，进一步夯实全市人民为率先建成全面小康社会团结奋斗的共同思想基础。把社会主义核心价值体系融入市民教育和精神文明建设全过程，贯穿于文化建设的各个方面，着力构建和谐文化。大力培育和弘扬创业创新创优精神，激励全体市民奋发向上、不断进取。深入开展公民道德实践活动，加强社会公德、职业道德、家庭美德、个人品德建设。巩固和扩大文明创建成果，进一步提高市民文明素质和社会文明程度。
     加快繁荣文化事业。建成市博物馆、大剧院、新图书馆并对外开放，启动建设市广电中心、体育中心、科技馆，实施青少年宫原址重建工程，进一步完善基层文化设施，构建较为完备的公共文化服务体系。推进市区数字电视整体平移、“村村通有线电视”和文化信息资源共享工程，办好2008中国李白诗歌节、第23届“江南之花”等文化活动，丰富城乡居民精神文化生活。开展以“全民健身与奥运同行”为主题的群众性体育健身活动，推进农民体育健身和社区体育健身工程，进一步提高竞技体育水平。
     积极推动文化创新。整合文化资源，加快推进文化体制改革，创新公益性文化事业单位体制机制，培育新型文化市场主体。制订文化产业发展规划，出台落实相关政策措施，推进洪滨丝画等市级文化产业示范基地、中国美术学院国际文化艺术交流中心和经纶文化传媒三期工程建设，促进文化产业加快发展。拍摄有关诗仙李白的影视作品，打造文化精品。加强文化和新闻出版市场管理，促进网络文化健康发展。积极开展对外文化交流，不断扩大城市文化影响力。
     保护和利用好文化遗产。开展第三次文物普查工作，制定当涂民歌、太平府铜壶工艺保护规划。加强对和尚桥铁矿开采等建设工程中的文物保护，实施朱然家族墓地保护及开发工程，启动建设烟墩山遗址公园，做好具有地方文化特色的馆藏文物修复工作。完成李白墓保护规划，加强工业遗址保护，推动城市历史文化的研究与利用。
     （八）切实加强和谐社会建设。高度关注和认真解决群众最关心、最直接、最现实的利益问题，深入实施30项民生工程，让改革发展成果更多地普惠于民，进一步提高全民生活质量。
     加快发展教育和卫生事业。合理配置公共教育资源，继续推进中小学布局调整，实施雨山路以南普通高中布点规划，启动义务教育阶段学校师资交流工作，关注留守及流动儿童教育与培养。完成特教学校新校区建设，启动二十中迁址建设工程，确保花园中学秋季全面建成。实施普通高中优质化战略，进一步提高农村高中阶段教育毛入学率。大力发展职业教育，建设职业教育实训基地，开展学分制试点和订单式培养，提高办学灵活性和教学质量。加快安工大东校区二期工程建设，独立设置马鞍山职业技术学院，确保河海大学闻天学院秋季招生。设立民办教育扶持奖励专项资金，推动民办职业教育和民办高等教育加快发展。深入实施《全民科学素质行动计划纲要》，大力提高劳动者科学文化素质。完善重大疾病预防控制、医疗急救和社区卫生服务体系，积极推进城乡爱国卫生工作，启动实施雨山区全国社区卫生服务适宜技术试点项目。以深化管理体制和运行机制改革为重点，进一步完善城乡医疗服务体系，提高服务质量和水平。稳定低生育水平，提倡婚前医学检查，提高出生人口素质，促进出生人口性别比趋于平衡。
     推进城乡统筹就业试点工作。深入落实《就业促进法》，完善就业再就业相关政策，实现以创业带动就业。建成当涂县农村劳动力专业市场，规范村级劳务公司运作。建立城乡统一的就业、失业登记与管理制度，探索制订农村劳动力失业标准。大力促进高校毕业生、农民创业就业，强化城乡困难群体的就业援助，实现“零就业家庭”动态为零。继续抓好各类培训，增强劳动者就业和创业能力。全年新增城镇就业2.1万人，其中下岗失业人员再就业8000人，援助困难群体再就业2000人，完成职业技能培训8000人；新增农村富余劳动力转移就业1.5万人。全面实施《劳动合同法》，进一步推进签约参保和企业用工登记备案制度，努力构建和谐劳动关系。
     完善城乡社会保障体系。做好养老保险省级统筹工作，继续推动有条件的企业建立年金制度。制定实施事业单位基本养老保险办法，探索建立城镇居民养老保险制度。健全失业保险促进就业联动机制，出台城镇个体工商户、灵活就业人员参加工伤、生育保险实施办法，推进马钢职工医疗及工伤、生育保险与全市并轨。完善被征地农民基本养老保障制度，积极推行新型农村养老保险制度，促进政策衔接，扩大覆盖范围。巩固完善新型农村合作医疗制度，提升参保农民医疗保障水平。强化社会保险扩面征缴和基金监管，确保各项社会保障待遇按时足额支付。提高城乡居民最低生活保障标准，完善困难家庭的医疗、教育救助体系，探索建立分层分类的最低生活保障制度，建立低保与就业再就业联动机制。加快推进乡镇敬老院改扩建步伐，提高农村五保集中供养率，新建市示范性老年公寓，继续大力做好老年人和残疾人工作。
     继续强化社会管理。加强社会治安综合治理工作，深入开展平安创建活动，健全社会治安防控体系，完善社区警务和农村警务工作，强化动态治安巡逻，做好社区矫正和刑释解教人员安置帮教工作。扩大法律援助覆盖面，保护困难群众和弱势群体合法权益。加强和谐社区建设，创新社区管理体制，做好第七届村委会换届选举工作。完善信访提前介入和督查督办机制，充分发挥人民调解制度的作用，妥善化解各种矛盾纠纷。加强流动人口服务和管理。深入推进民族、宗教、外事、侨务和对台工作。全面落实安全生产责任制，严格安全生产监管，严防重特大事故发生。建成启用国家一级气象站，完善气象、地震防灾减灾服务体系。贯彻实施《突发事件应对法》，及时应对和妥善处置突发公共事件。加强市场引导、监管，确保市场供应和商品质量安全。进一步疏导价格矛盾，保持物价总体水平基本稳定。加强国防动员、民兵预备役和人民防空工作，大力落实全民国防教育，增强全民国防意识。支持驻马解放军和武警部队建设，深入开展双拥共建活动，巩固和发展军政军民团结。
     （九）深入推进政府服务创新。以提高政治业务素质为根本，以转变政府职能为核心，以规范行政权力为重点，以提升行政效能为目标，进一步加强政府自身建设。
     大兴学习调研之风。按照建设学习型政府的要求，深入学习宣传贯彻党的十七大精神，用中国特色社会主义理论体系武装头脑，进一步解放思想、创新观念，广泛开展实践科学发展观活动，切实提高运用理论创新引领实践创新、解决实际问题的能力。政府系统全体工作人员要自觉学习现代科学文化知识，加快知识更新，优化知识结构，不断丰富做好政府工作的知识储备。要坚持理论联系实际，加强对事关发展全局战略性、前瞻性问题的??施，更加有力地推动经济社会又好又快发展。
     深化行政管理体制改革。优化经济调节和市场监管，强化社会管理和公共服务，促进政府职能的转变。完善公共财政体制，加快国库集中支付制度改革，规范政府采购行为。进一步理顺市与区财政收入分配关系，不断健全区级功能，逐步实施支出项目效益审计，健全财政支出绩效评价机制。健全行政服务中心功能，推进“两集中、两到位”工作，积极探索相对集中行政审批权，加快推进网上审批。加强电子政务建设，建立全市电子政务办公应用平台，逐步实现办公电子化。
     全面推进依法行政和民主管理。自觉接受人大及其常委会的监督，积极支持政协履行职能，扎实做好人大代表议案和政协提案办理工作，主动听取各民主党派、工商联和无党派人士的意见和建议，充分发挥各人民团体的作用。健全政府重大决策专家咨询、重大事项社会公示和听证制度，进一步扩大政府信息公开范围。认真做好第二次全国经济普查工作，积极推进档案信息化建设，夯实行政管理工作基础。进一步发挥政府层级监督作用，加强行政复议工作，健全行政执法责任制，出台落实依法行政监督办法，提高行政工作人员依法行政能力和水平，促进严格执法、高效执法、公正执法、文明执法。
     大力加强作风建设和廉政建设。加强理想信念和思想道德建设，努力使政府工作人员成为科学发展观的忠实执行者、社会主义荣辱观的自觉实践者、社会和谐的积极促进者。政府全体工作人员要始终保持奋发有为、奋力争先的精神状态和求真务实的工作作风，勤于思考，勇于开拓，敢于负责，快速落实，不断开创各项工作新局面。进一步深化机关效能建设，加大目标考核奖惩力度，完善重大事项和重点工作督促落实机制，确保政令畅通。规范公务接待和后勤保障行为，降低行政成本，努力建设节约型政府。完善教育、制度、监督并重的惩治和预防腐败体系，严格执行廉洁自律各项规定，严肃查处商业贿赂等违法违纪行为，坚决纠正损害群众利益的不正之风，树立清正廉洁的政府形象。
     各位代表，马鞍山已步入全面跨越、整体提升的崭新发展阶段。站在新的历史起点上，我们倍感使命光荣、责任重大，倍感机遇可贵、形势催人。让我们高举中国特色社会主义伟大旗帜，紧密团结在以胡锦涛同志为总书记的党中央周围，以邓小平理论和“三个代表”重要思想为指导，深入贯彻落实科学发展观，在省委、省政府和市委的领导下，依靠全市人民，凝聚各方力量，继往开来，顽强拼搏，为率先建成全面小康社会、加速构建和谐马鞍山、创造人民美好新生活而努力奋斗！
